# Tribological Properties of Aluminium Alloy Composites Reinforced with Multi-Layer Graphene—The Influence of Spark Plasma Texturing Process

**DOI:** 10.3390/ma10080928

**Published:** 2017-08-10

**Authors:** Marek Kostecki, Jarosław Woźniak, Tomasz Cygan, Mateusz Petrus, Andrzej Olszyna

**Affiliations:** Faculty of Materials Science and Engineering, Warsaw University of Technology, Wołoska St. 141, 02-507 Warsaw, Poland; j.wozniak@inmat.pw.edu.pl (J.W.); t.cygan@inmat.pw.edu.pl (T.C.); mateusz.petrus@inmat.pw.edu.pl (M.P.); aolszyna@inmat.pw.edu.pl (A.O.)

**Keywords:** aluminium matrix composites, multilayer graphene, SPS sintering, tribological properties

## Abstract

Self-lubricating composites are designed to obtain materials that reduce energy consumption, improve heat dissipation between moving bodies, and eliminate the need for external lubricants. The use of a solid lubricant in bulk composite material always involves a significant reduction in its mechanical properties, which is usually not an optimal solution. The growing interest in multilayer graphene (MLG), characterised by interesting properties as a component of composites, encouraged the authors to use it as an alternative solid lubricant in aluminium matrix composites instead of graphite. Aluminium alloy 6061 matrix composite reinforced with 2–15 vol % of MLG were synthesised by the spark plasma sintering process (SPS) and its modification, spark plasma texturing (SPT), involving deformation of the pre-sintered body in a larger diameter matrix. It was found that the application of the SPT method improves the density and hardness of the composites, resulting in improved tribological properties, particularly in the higher load regime.

## 1. Introduction

Composites based on aluminium and its alloys, compared to unreinforced material, offer a number of attractive properties, such as greater strength, improved stiffness, reduced density (weight), and improved temperature properties (creep resistance). As such, engineers are considering them more and more often, and the number of commercially available materials is constantly increasing [1]. The most important applications are related to the automotive and aerospace industries, where a significant increase in durability is needed while maintaining low weight for structural components [2]. Composites based on boron fibre–reinforced aluminium alloys have been known since the 1960s, when they were first used in aerial construction [3]. Since then, a number of materials, mainly ceramic, such as SiC, Al_2_O_3_, B_4_C, Si_3_N_4_, AlN, TiC, TiB_2_, and TiO_2_, have been proposed to alter the properties of the aluminium matrix, often with very different morphology, ranging from equilibrium particles to whiskers or fibre forms [4]. Initially, the graphite fibres were used to reinforce polymer matrix composites because of the reactivity of carbon with magnesium and aluminium. In the 90s, hybrid composites emerged that, besides increased strength or creep resistance due to additional components, were characterised by increased tribological properties resulting from the addition of graphite solid lubricant [5,6]. The success achieved in these materials involved the use of completely new manufacturing methods to which we can include powder metallurgy [7,8].

The use of solid lubricants as an integral component of the composite allows the composite to achieve a self-lubricating effect. This process is the result of the formation of a thin film between the surface of the material and the parts cooperating with it. This allows for significant reduction in the coefficient of friction (COF) due to the replacement of the external friction of the solids by the internal friction of the particulate lubricants [9]. Rohatgi et al. [10] first introduced graphite as a solid lubricant in aluminium matrices by casting routes. Much work has been done to modify and optimise the properties of such composites. Unfortunately, soft graphite or MoS_2_ particles commonly used in micron size are responsible for the simultaneous deterioration of mechanical properties such as hardness, modulus, and strength. Moreover, those particles are susceptible to the formation of defects and cracks, which results in premature failure in mechanical testing [11].

The single layer graphene cannot be the proper reinforcement for self-lubricating composites because of the absence of a layered structure. Therefore, the use of multilayer graphene may be the best way to modify the composite structure [12]. The presence of several to dozens of layers allows the film to be made, while thin flakes will not significantly reduce mechanical properties. A number of authors have attempted to produce such composites with good results. Although different authors have used morphologically differentiated multilayer graphene, in each of these cases, the process has improved mechanical properties such as tensile strength, [13,14,15,16] yield strength, [17,18,19], and compressive strength [20]. Moreover, the improvement in performance is even better than in the case of previously used carbon nanotubes [11]. The phenomenon can be explained as a results of 2D crystals’ good adhesion to the matrix and increased interfacial surface area, which restrains dislocation and crack propagation during tensile tests.

An important fact confirmed experimentally is the small volume fraction of flakes required for improvement of these properties, which in most cases does not exceed 1%. After exceeding the barrier of 1% in weight, as in case of large particles, there is a decrease in mechanical properties related with strong agglomeration of flakes into 3D forms. This is a typical tendency of powder composites. Similar dependencies are associated with agglomeration with high MLG volume and were observed by authors for Al_2_O_3_ ceramics composites [21].

Far fewer literary references can be found about the tribological properties of aluminium matrix composites with MLG participation [18,19]. However they point to the possibility of improving tribological properties and are difficult to compare because of different way of manufacture and different testing methods. Additionally for all the studied materials, graphene content does not exceed 3% by volume. Research conducted in the 70s’ [22] denote that the use of specific morphology of solid lubricant particles may be important for the friction process. Considerations concerning the geometry of the grease indicate that the ratio of the surface area of the basal planes to the edges of the particles is significant. Increasing this coefficient allows for a significant reduction in friction resistance. The expected effect of the presence of the MLG in the composite structure is a decrease in the coefficient of friction and a positive impact on the mechanical properties. It can be inferred that this will also contribute to a reduction in wear. However, the mechanisms responsible for wear processes are more complex.

Authors in previous studies [23] have attempted to explain the effect of both the type of solid lubricant (multilayer graphene and molybdenum disulphide) and the amount of lubricant needed to produce a friction film and showed that the required amount of MLG needed to improve the tribological properties (COF and wear) is about 10%. This is far beyond the scope of the guarantee of increased mechanical properties. At the same time, it was indicated that the geometry of the flakes and aluminium powder particles can influence their specific orientation in the microstructure, which indirectly causes the applied axial pressure synthesis method.

Based on previous research, we have attempted to change the manufacturing parameters by modifying the SPS process in the current considerations. The proposed change aims to obtain a composite characterised by better dispersion of MLG and their further orientation and improved mechanical properties. The applied modification is a process previously performed for other types of materials [24,25] and has been called Spark Plasma Texturing (SPT) because of the specific microstructure characterised by its possible texture. The purpose of the SPT process is to perform SPS sintering with a simultaneous dimension change, which consists of plastic deformation of the pre-sintered body in a die of larger diameter than the compact. As a result, we get a sinter with a larger diameter and less height. The aim of the authors was to examine the influence of the obtained microstructure of composites with multilayer graphene on the tribological properties of the AA6061 matrix.

## 2. Materials and Methods

**Figure 1:**
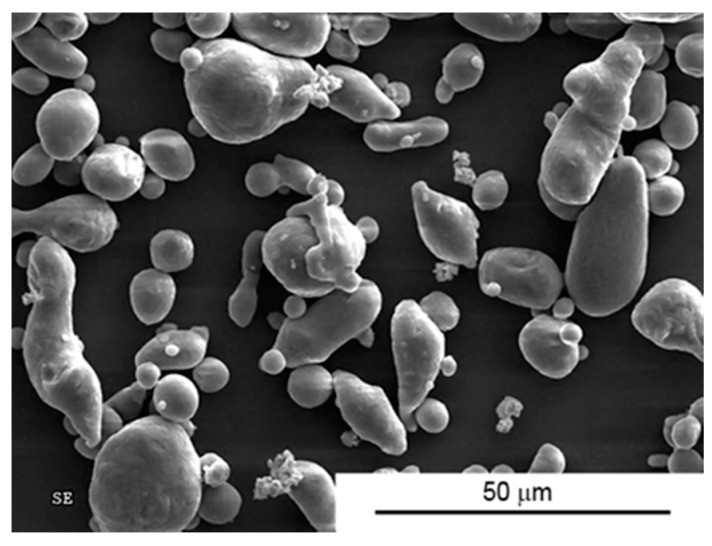
Morphology of AA6061 powder.

In order to produce the composites, Alpoco AA6061 commercial aluminium powder was used as the matrix and multi-layered graphene flakes as a solid lubricant. Graphene powder with the trade name Gn(12) comes from Graphene Supermarket (Calverton, New York, NY, USA). The morphology of the aluminium powder particles is shown in Figure 1. Based on the image below, it can be seen that the particles have different shapes. The mean diameter of a particle (d_2_) is 10.9 μm. The coefficient of elongation (μ) for the particles is 1.46, which indicates a certain deviation from the spherical shape.

**Figure 2:**
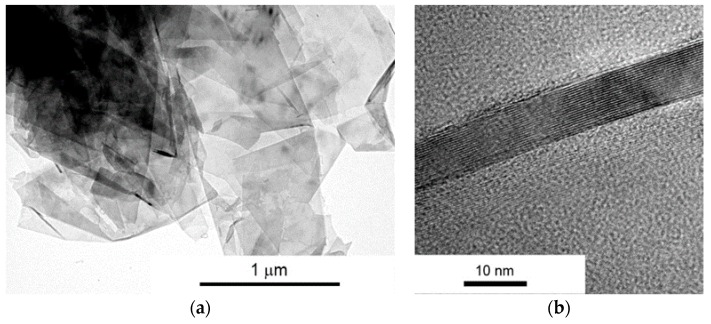
(**a**) STEM and (**b**) HRTEM micrographs of Gn(12) multilayer graphene (MLG) powder.

Figure 2 shows the morphology and transmission image of a cross section of the flake. The name Gn(12) denotes that the average thickness of one flake is 12 nm (Figure 2b), about 30 single graphene layers. It was examined that the average lateral size is approximately 4.5 μm.

**Figure 3:**
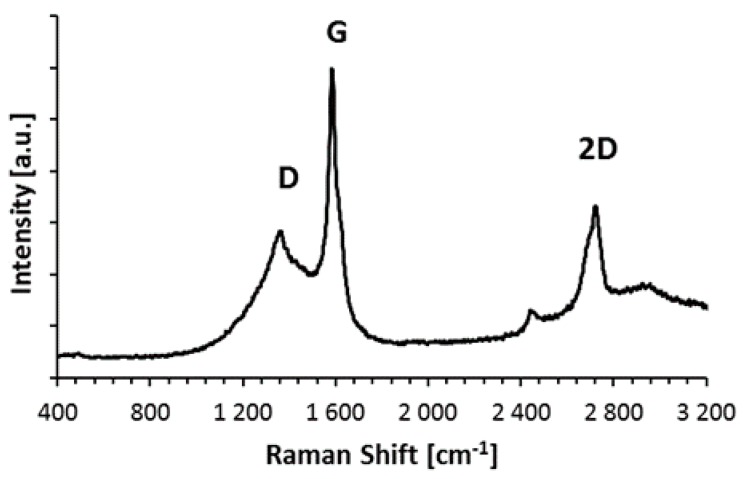
Raman spectra of Gn(12) MLG powder.

The Raman spectra of the graphene is presented. Based on the spectrum (Figure 3), the I_2D_/I_G_ intensity ratio is set at about 0.60. This value and peak shape is typical for multi-layer graphene [26].

The first step in the manufacturing process was to prepare the powders’ specific chemical composition i.e., (0, 2, 5, 10, 15) vol % of MLG. Graphene content was identical for samples prepared in the SPS and SPT methods. Then powders were mixed in a horizontal rotary mill. The homogenizing parameters used were: mixing time 12 h and rotational speed 300 rpm were previously optimized for minimizing agglomeration. Zirconia beads (2 mm in diameter) in a ratio of 3:1 powder weight was used. Mixes were held in a suspension of isopropyl alcohol. The resulting slurry was dried and then granulated on # 0.3 mm sieves.

**Figure 4:**
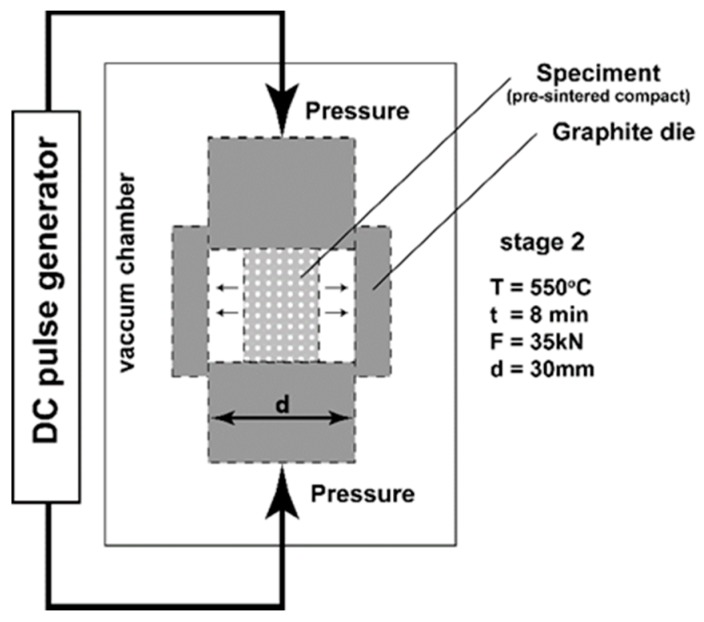
SPT processing scheme.

The last step was the consolidation of the powder - in the first by the SPS method, the second in the SPT method in two stages. The parameters of the SPS and SPT process were selected on the basis of optimisation work carried out for the selected Al/10 vol % MLG composition and pure Al alloy powder. The intention of the authors was to obtain the highest relative density of composites. These parameters are shown in Table 1. Figure 4 shows schematically the SPT process and its parameters.

The obtained sinters were mechanically grinded and polished down to a grit size of 0.2 mm and subjected to further investigations. Fundamental properties of the obtained materials, such as density (Ultrapycnometer 1000 helium pycnometer Quantachrome Instruments), and hardness (Vickers Hardness Tester FV-700e, Future-Tech, Kawasaki-City, Japan) were measured. XRD analysis was performed to determine the orientation of graphene flakes. These tests were performed with the same parameters on two identical volumes of composites with 10 vol % Gn(12) after the SPS and SPT process. For this purpose, a Bruker D8 Discover (Germany) X-ray diffractometer was used, working with a 0.1540 nm wavelength Cu tube. The test was carried out at room temperature. The angular range was 10°–120°, and the counting time was 5 s. Quantitative texture analysis was based on three incomplete pole figures: (111), (200), and (220). The measurements were made using a Bruker D8 Discover X-ray diffractometer filtered Co Kα radiation. Based on the measured pole figures for each sample, the orientation distribution functions were obtained and quantitative ratios of the major texture components were calculated.

Tribological tests were performed by a ball-on-disc type tribometer (T-21 tribotester, ITeE-PIB Radom, Poland) at room temperature (23 °C) and humidity 35%, using 0.1 m/s sliding speed and normal loads from 1 to 10 N. The tests were carried out for 1000 revolutions. As a counterpart, 100Cr6 steel (10 mm in diameter) balls were selected. The coefficient of friction and wear ratio were determined according to the requirements defined in ASTM G 99-05 [27]. Each test was repeated at least three times per sample, and the average measurements value is shown in the graph. The worn surfaces, microstructure of composites, and substrates morphology were observed using scanning electron microscopes (Hitachi S3500 and S5500, Japan). Raman analysis was made using a Nicolet Almega XRD dispersive Raman spectrometer (Thermo Fisher Scientific, Waltham, MA, USA) (laser with an excitation wavelength of 532 nm), and at least three scans for each sample were performed.

## 3. Results and Discussion

**Figure 5:**
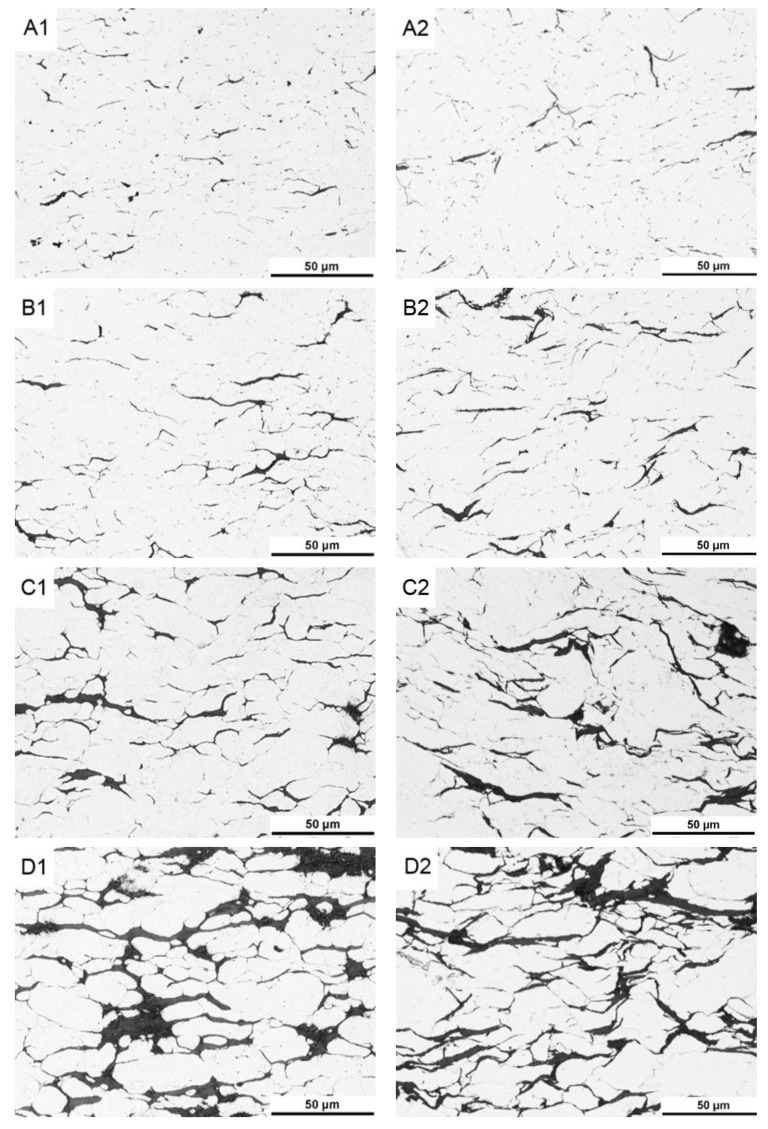
Microstructure of AA6061/MLG composites obtain in SPS (**A1**—2 vol %; **B1**—5 vol %; **C1**—10 vol %; **D1**—15 vol %) and SPT (**A2**—2 vol %; **B2**—5 vol %; **C2**—10 vol %; **D2**—15 vol %) process.

The most important differences in materials produced by SPS and SPT methods should relate to the dispersion of MLG particles in the composite volume. Figure 5 shows images of microstructures of composites obtained in various processes, indicating the cross sections parallel to the direction of acting force (perpendicular to the surface of the die stamp). Dark areas are places of occurrence of carbon (MLG) agglomerate, also thin aluminium powder particle boundaries are partly visible. Many of the fine pores and phases of Mg_x_Si_y_ identified earlier in the XRD study are visible as dispersed dark objects in the middle of light areas (aluminium powder particles). The apparently fibrous structure of graphene flakes indicates their orientation in the volume of the composite. They are arranged in planes perpendicular to the direction of the operating force of compression, in a manner typical of powdered composites with particles of morphology of the flakes.

**Figure 6:**
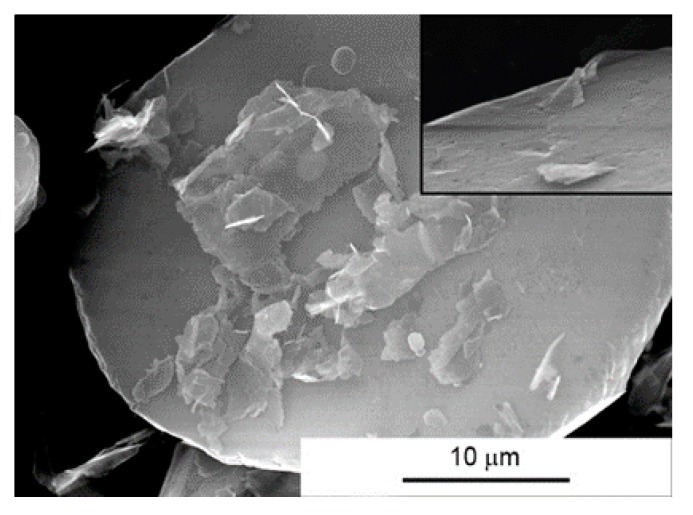
Morphology of AA6061/MLG powder mixture after homogenization.

**Figure 7:**
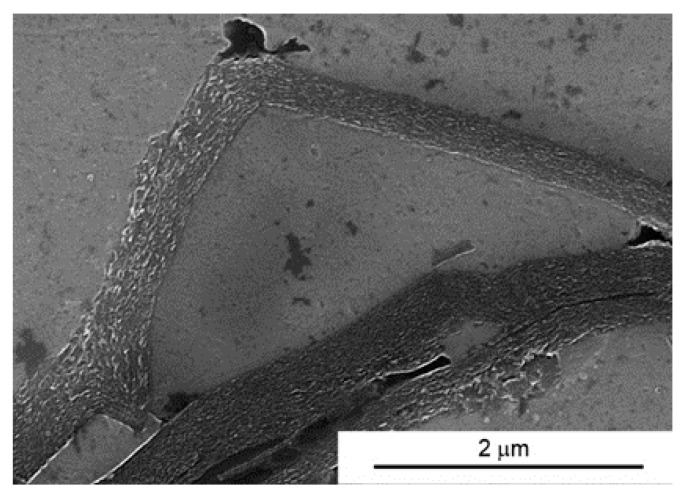
Microstructure of AA6061/10 vol % MLG composite obtain in SPT process.

At the homogenization stage of the suspension, the graphene flakes “stick” to slightly flattened aluminium powder particles (Figure 6). This causes a specific orientation of the graphene in the composite fixed by axial pressure in the SPS process. When some changes in the deformation scheme occur in the SPT process, areas rich in agglomerates of multi-layered graphene flakes become displacing and delaminating. Stereological analysis on cross sections indicates a significant reduction in thickness of graphene flakes agglomerates. In the composites with 15% volume, the average thickness of agglomerates changes from 5.4 μm (SPS) to 3.6 μm (SPT). Present in SPS composites, the large pores caused by the presence of MLG agglomerates (Figure 5D1) disappear almost completely in SPT composites. At the same time, this process causes the changes of the graphene plane’s orientation from an axis perpendicular to the force direction, as a result of the free movement of the particles in the larger matrix until the volume is completely filled. Only small amounts of pores resulting from non-uniform delamination of MLG flakes remain in the structure, as shown in Figure 7.

**Figure 8:**
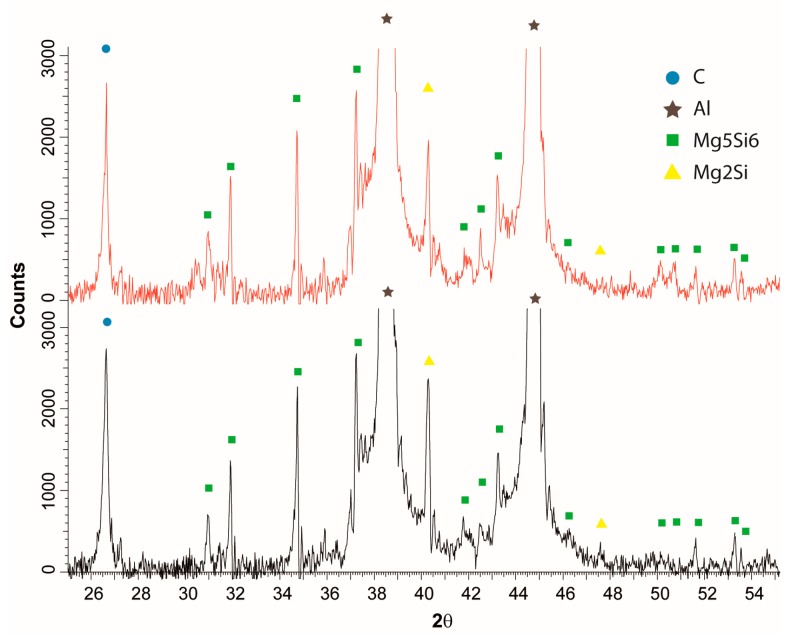
Diffraction pattern of SPS (red line) and SPT (black line) AA6061/10 vol % MLG composites.

Due to the expected and observed displacements of MLG during the texturing process, diffraction tests for standard and textured samples were performed. A comparison of the intensity of certain carbon peaks should indicate a change in the orientation of the flakes in the volume of the composite. In order to ensure comparable measurement conditions, two sections were prepared for each composition, and samples of identical geometry and volume were cut. Figure 8 presents diffraction patterns for SPS and SPT samples recorded on similar cross sections. The record indicates the significant conformity of the identified carbon to the graphite. There was no difference in peak intensity from plane (002), 2θ = 26.67, indicating a similar orientation of the flakes on the examined sections. Diffraction analysis also confirms the absence of aluminium carbides that could form during the sintering process.

**Figure 9:**
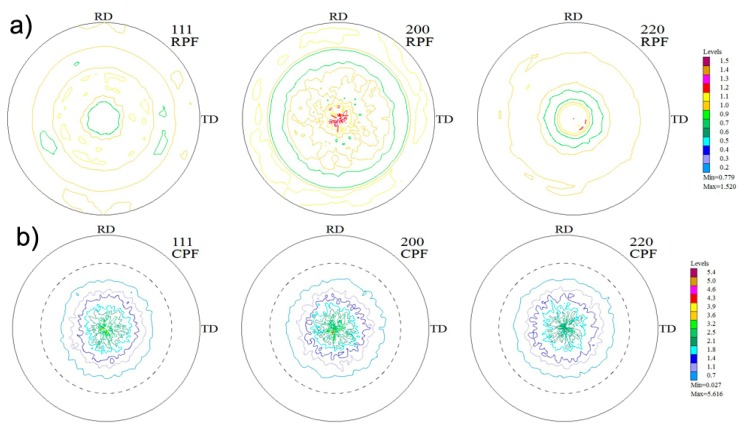
Pole figures: calculated (**a**) and experimental (**b**)—(111), (200), (220) for AA6061/10 vol % MLG composites obtain in SPT process.

Texture analysis for aluminium grain was made for four samples with a varying volume content of multilayer graphene at their midpoint and at the edges. The Figure 9 shows the exemplary pole figures (experimental and computational) for the Al/10 vol % MLG composite. On the basis of the research, it can be stated that in all cases, the resulting texture for Al is poorly shaped, which may prove to be low value on the scale. It is also not possible to find the correlation of changes in texture component intensity depending on the contribution of graphene in the matrix.

**Figure 10:**
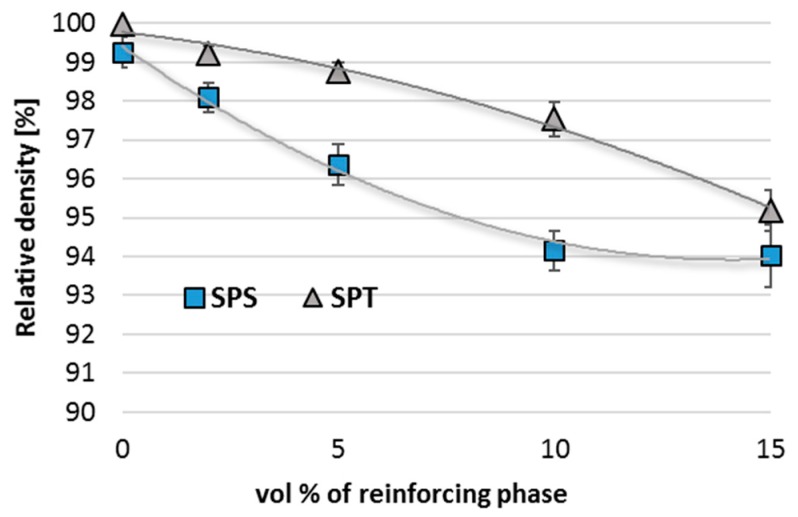
Relative density of AA6061/MLG composites obtain in SPS and SPT process.

The previously observed absence of large pores within the agglomerates on the microstructure after the SPT process has some consequences in a significant increase in density. Density increases throughout the range of compositions, and in the case of 10 vol % of graphene, we can observe an increase in relative density by 3.3%. (Figure 10).

**Figure 11:**
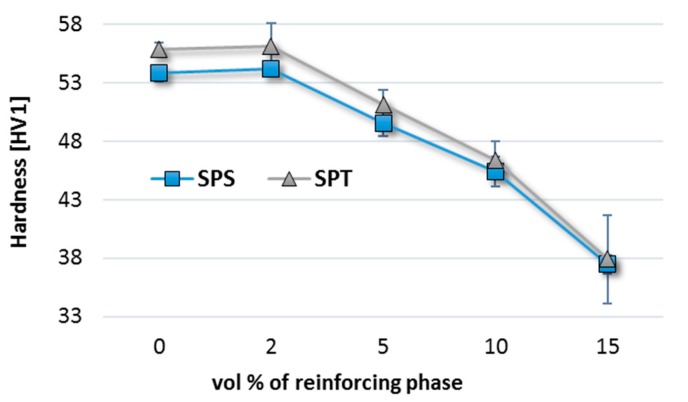
Vickers hardness of AA6061/MLG composites obtain in SPS and SPT process.

The density factor is correlated with the hardness test results. However, the hardness increase is not spectacular. SPT composites with a small volume fraction of multilayer graphene (2%) have a slightly higher hardness than samples without graphene addition (Figure 11). With the increase in the amount of flakes, the composite hardness decreases to 37 HV1 for the composite with 15 vol % MLG versus 56 HV1 for the composite with 2 vol % MLG, but the higher hardness of the composites produced in the SPT process is visible in the whole range of compositions.

**Figure 12:**
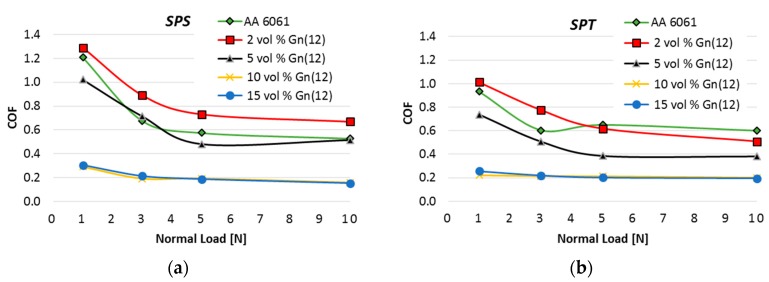
Coefficient of friction of AA6061/MLG composites obtain in SPS (**a**) and SPT (**b**) process.

An analysis of the tribological properties was carried out, taking into account the coefficient of friction, wear rate, and the nature of worn surface. Figure 12a,b shows the results of the friction coefficient related to the friction load.

Comparing the diagrams (Figure 12), we can say that changes in the consolidation process resulted in a relatively large reduction in friction coefficient for materials with a small volume fraction of MLG. The most likely mechanism of improving COF is associated with an increase in hardness. For example COF of composites with a 2 vol % of MLG decreased from 1.3 (SPS) to 1 (SPT). Hardness has a stronger effect on the decrease of COF at low loads of 1 and 3N although it is also noticeable for 5 N load.

It should be noted, however, that regardless of the consolidation method, the small addition of MLG composites (2 vol %) has a higher COF than unreinforced sinters. The thinnest agglomerates observed in these composites do not contribute to create lasting tribofilm on the surface. Furthermore, in the shear stress regime, there is a good chance to form monolayer graphene that, due to the adhesive bonding to the aluminium surface, will form a conglomerate of considerable hardness. As a result of the cyclical impact of contact stresses (surface fatigue), the conglomerate will be detached and, as a hard particle, will increase friction resistance and contribute to increased wear of the composite. This is confirmed by the results of wear presented later in the article.

For composites with high volume fraction 10–15 vol % MLG, an interesting effect of load independence on the COF was obtained, and the SPT process has only slightly intensified this relation. In the range of 1 to 10 N, COF is maintained at a constant value close to 0.2. The dominant mechanism of COF improvement is due to the presence of self-healing tribofilm. The general tendency of the decrease in frictional resistance with an increasing load was retained for all composites in given conditions.

**Figure 13:**
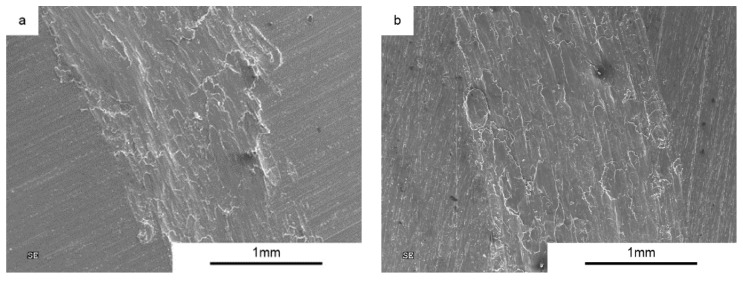
Worn Surface morphology for AA6061 load 1N (**a**) SPS and (**b**) SPT.

In the following images (Figure 13), we can see the nature of the wear for AA6061 sinters. The type of wear can be described as adhesion with significant plastic deformation in the contact zone. The nature of the friction process does not change significantly for samples produced by the SPT method, although much less plastic deformation is visible.

**Figure 14:**
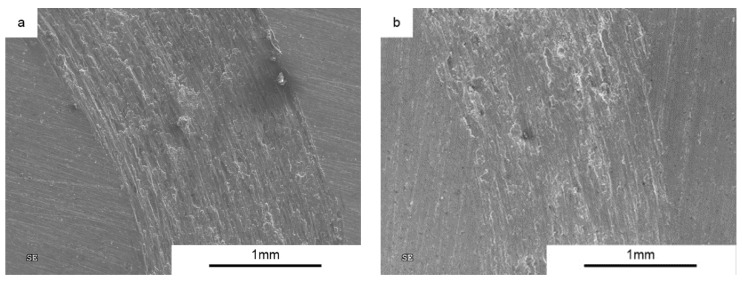
Worn Surface morphology for AA6061 + 2 vol % MLG load 1N (**a**) SPS and (**b**) SPT.

**Figure 15:**
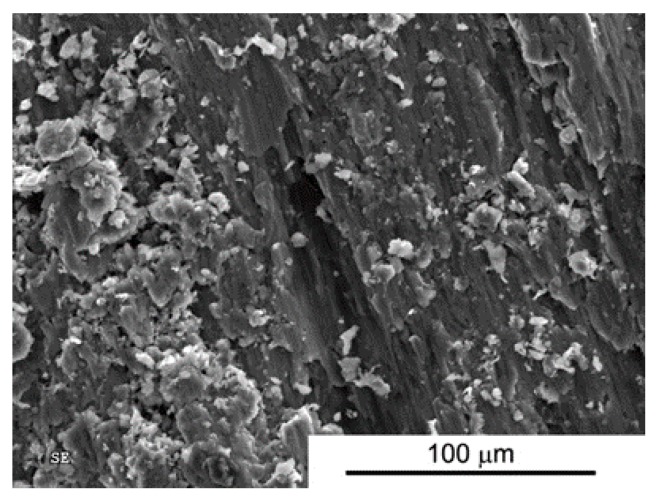
Worn Surface morphology for SPS AA6061+2 vol % MLG load 5N.

An addition of 2 vol % MLG changes the nature of the wear process (Figure 14). Numerous grooves, delamination, and a large number of flakes and round particles (wear products) are evident, suggesting participation of abrasive wear mechanism (Figure 15).

**Figure 16:**
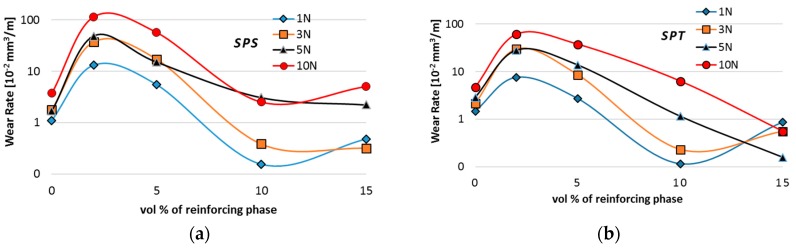
Wear rate of AA6061/MLG composites obtain in SPS (**a**) and SPT (**b**) process.

For all composites, we observe typical situations of increased wear as the function of increasing load (Figure 16). A high increase in the wear for composites with additions of 2% and 5 vol % MLG confirms the previously proposed theory of the presence of Al/graphene conglomerates. Figure 15 shows the presence of fine particles of approximately 5–15 μm in average, uniformly covering the worn surface.

**Figure 17:**
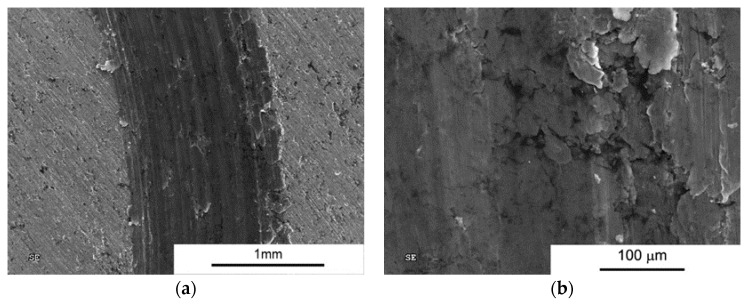
Worn Surface morphology for AA 6061 + 10 vol % MLG load 10N SPS. (**a**) Wear trace image; and (**b**) close-up view.

**Figure 18:**
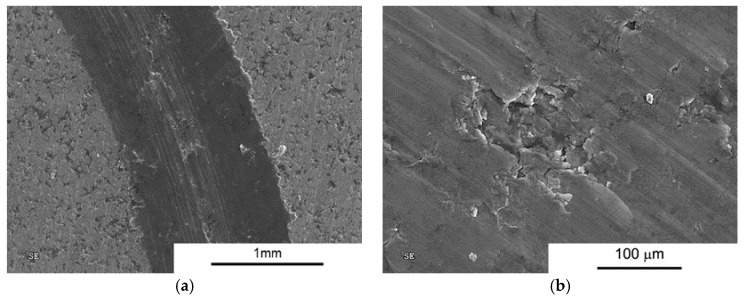
Worn Surface morphology for AA 6061 + 15 vol % MLG load 10N SPS. (**a**) Wear trace image; and (**b**) close-up view.

However, when analysing the wear test results, particular attention should be paid to composites subject to wear processes at higher loads. In the case of the SPS process, wear reduction compared to AA6061 sinter was observed only for composites with high volume fraction of MLG (10% and 15%) and only for low loads. Analysis of wear trace marks indicates the significant effect of porosity on the wear process (Figure 17 and Figure 18). We can see numerous cracks beginning at the edge of the pores that initiate the process of peeling off larger pieces of material in mechanisms of fatigue and abrasion.

**Figure 19:**
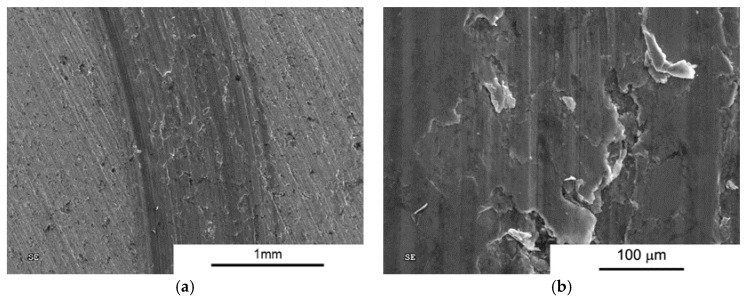
Worn Surface morphology for AA 6061 + 10 vol % MLG—load 10N SPT. (**a**) Wear trace image; and (**b**) close-up view.

**Figure 20:**
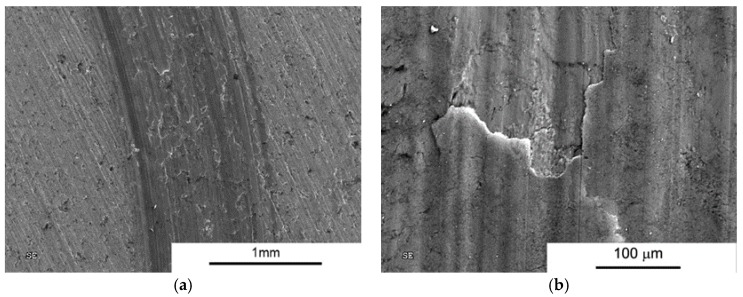
Worn Surface morphology for AA 6061 + 15 vol % MLG—load 10N SPT. (**a**) Wear trace image; and (**b**) close-up view.

The use of the SPT technique reduced the thickness of MLG agglomerates and the number of pores within these agglomerates. At the same time, the total surface of particle boundaries occupied by the solid lubricant increased. This makes it easier to access the carbon flakes during the friction process and to maintain the process of creating a tribofilm. The observed worn surfaces are more homogeneous. Direct abrasive damage is not visible in the plastic deformation zone. We can observe spalling with local interruption of the lubricant film especially for composites containing 10 and 15 vol % of MLG (Figure 19 and Figure 20).

## 4. Conclusions

The addition of above 2 vol % of multilayer graphene to an aluminium alloy matrix causes composite porosity and a decrease in their mechanical properties (such as hardness) as a result of strong agglomeration in the form of relatively large flakes in thickness and lateral size. In the case of a small volume fraction of graphene, we observe a small increase in hardness that can be caused by two factors: limitation of grain size growth during the sintering process, and blocking of dislocations motion at the boundaries of aluminium grains. The use of MLG as an additive in aluminium matrix composites significantly improves their tribological properties, friction coefficients, and wear rate, especially for composites with 10 vol % and 15 vol % graphene. With the increase of graphene content in composites, the change in the wear mechanism is observed. The abrasive nature of wear disappears after the addition of about 10 vol % MLG and self-healing tribofilm appears.

The use of the SPT method makes it possible to produce composites with higher relative density compared to SPS materials. The SPT process results in a more homogeneous structure and better dispersion of graphene, which results in a reduction in the number of pores in the agglomerates and an increase in the surface covered with flakes on which a tribofilm is formed.

The SPT method may be a compromise between improving tribological properties and retaining the good mechanical properties of the composites. The process of optimisation of the composite properties proposed in this paper should take into account further work to ensure the anisotropic microstructure, the directional dispersion of the flakes, and the reduction of their thickness. This can be achieved using a higher degree of processing in the SPT method.

## Figures and Tables

**Table 1:** Spark plasma sintering (SPS) and spark plasma texturing (SPT) process parameters.

	SPS	SPT	
		Stage 1	Stage 2
**Temperature (°C)**	550	300	550
**Pressing force (kN)**	35	3	35
**Pressing time (min)**	2	2	8
**Atmosphere**	Argon	Argon	Argon
**Heating rate (°C/min)**	150	150	100
**Die diameter (mm)**	30	20	30
